# Systematic Research on the Transport of Ball-Milled Biochar in Saturated Porous Media: Effect of Humic Acid, Ionic Strength, and Cation Types

**DOI:** 10.3390/nano12060988

**Published:** 2022-03-17

**Authors:** Gang Cao, Jiachang Qiao, Juehao Ai, Shuaiqi Ning, Huimin Sun, Menghua Chen, Lin Zhao, Guilong Zhang, Fei Lian

**Affiliations:** 1 College of Natural Resources and Environment, Northwest A&F University, Xianyang 712100, China; caogang0314@nwafu.edu.cn (G.C.); qiaojc@nwafu.edu.cn (J.Q.); aijuehao@nwafu.edu.cn (J.A.); ningshuaiqi@nwafu.edu.cn (S.N.); chenmenghua@nwafu.edu.cn (M.C.); 2 Key Laboratory of Plant Nutrition and the Agri-Environment in Northwest China, Ministry of Agriculture, Xianyang 712100, China; 3 Shaanxi Provincial Research Academy of Environmental Sciences, Xi’an 710061, China; zhaolin_1204@163.com; 4 Agro-Environmental Protection Institute, Ministry of Agriculture and Rural Affairs of the People’s Republic of China, Tianjin 300191, China; 5 School of Energy and Environmental Engineering, Hebei University of Technology, Tianjin 300401, China; lianfei2000@126.com

**Keywords:** transport, ball-milled biochar, humic acid, cations, porous media

## Abstract

Ball-milled biochar (BMBC) is a typical engineering material that has promising application prospects in remediating contaminated soil and water. It is fundamental to rate the transport behaviors of BMBC in the underground environment before extensive use. In this study, the effects of the ubiquitous cations (Na^+^, Mg^2+^, and Al^3+^) and model organic matter (humic acid) on the transport of BMBC were investigated using laboratory column experiments. The results demonstrated the facilitated effect of HA on the transport of BMBC due to the negatively charged surface and steric effect under neutral conditions. HA and ionic strength manifested an antagonistic effect on the transport of BMBC, where the presence of one could weaken the effect from the other. We also found the charge reversal of the BMBC surface in the presence of Mg^2+^, thus enhancing the deposition of BMBC onto the medium surface. On the other hand, the charge reversal from Al^3+^-coupled acid conditions led to the restabilization and transport of BMBC in porous media. Therefore, the rational usage of BMBC is indispensable and more attention should be paid to the composition and change in underground water that might facilitate the transport of BMBC and thus lead to negative environmental implications.

## 1. Introduction

The rational rethinking of agricultural waste recycling and carbon sequestration for reducing greenhouse gas emissions contributed to the discovery and application of biochar [1,2,3]. Nowadays, biochar has been widely applied in the agricultural and environmental fields with a high performance–price ratio. In particular, biochar is an important soil amendment in the improvement of physical and chemical properties of the soil [4,5,6,7], and a novel adsorbent that has a relatively strong adsorption capacity to heavy metals or organic pollutants through the mechanisms of complex formation, electrostatic behavior, ion exchange, etc. [8,9,10]. In practice, biochar has usually been directly applied and released into the soil and thus might lead to dust pollution or the invasion of fine particles into the underground environment [11,12]. Moreover, the strong adsorption capacity of biochar is a double-edged sword and brings the concern about transport behaviors of biochar particles that is important for the ecological environment and human health. Therefore, the investigation of biochar transport behaviors is an urgent subject that needs to be explored.

Modified biochar, which has expanded the application scenarios of biochar materials, is generally obtained from pristine biochar after simple treatments [9,13]. Among them, ball milling has been proven to be an environmentally friendly engineering method by applying the theory of mechanochemistry, which greatly reduced the particle size, increased the specific surface area, and exposed more active edge sites of biochar, thus improving its adsorption ability [14,15,16]. Over the past few years, a considerable number of studies have emerged that have indicated that the adsorption capacity of ball-milled biochar (BMBC) for heavy metals (e.g., Pb, Cd, Ni, Hg), dyes (methylene blue, methyl orange, rhodamine b, etc.), and other organic contaminants (antibiotic, pesticide, etc.) from the liquid phase environment has significantly increased compared to the pristine biochar [16,17,18,19,20,21,22,23]. The fine BMBC particles fulfill the facilitated transport requirements [24]: (1) with high affinity to pollutants, (2) with high mobility or ability to be mobilized either before or after the sorption, and (3) the ability of the compound of particles and pollutants to be transported in porous media. BMBC therefore might facilitate the transport of contaminants in the underground environment. On the other hand, BMBC itself and the synergistic effect of BMBC and pollutants are biotoxic to soil microbes. To a certain extent, the toxicity of BMBC might be stronger than those of other carbon-based nanomaterials (e.g., graphene oxide and multi-walled carbon nanotubes) [25,26]. 

The application of BMBC also inevitably causes a dispersion of BMBC particles into the environment. Obviously, people are not yet ready for that before understanding well its environmental effects. As is known, the fine biochar particles that are mostly generated from pore collapse and matrix fracture (these processes also occur in the ball milling process) have been intentionally or unintentionally released into the soil environment [11,12]. Previous studies have reported that solution chemistry (e.g., pH, ionic strength, cation types, organic matter, surfactants) [27,28,29,30,31] played a significant role in the transport of biochar due to the state of biochar stability in the aquatic environment [11,32,33]. The solution chemistry conditions of groundwater, however, are regularly influenced by rainwater, irrigation, fertilization, or artificial groundwater recharge, which lead to the perturbation of organic matter and metal cations such as Na^+^, K^+^, Ca^2+^, Mg^2+^, and Al^3+^ in groundwater [30]. Furthermore, the organic matter and these cations routinely exist at the same time and place, and significantly affect the transport and fate of biochar particles [29,34,35]. Though a lot is known about biochar, our understanding of the transport of BMBC is largely inadequate. 

Humic acid is widely found in groundwater and soil environments and is usually chosen as the model of organic matter [36]. The packed column has been widely applied in laboratory investigations for its efficiency and convenience [37]. To our limited cognition, there is still a knowledge gap in how the interactions between cation types and humic acid influence the transport of BMBC. Here, in this study, the prepared and characterized BMBC was subjected to a series of column experiments to systematically investigate the transport behavior of BMBC in the saturated porous media. Our specific objectives are: (1) to confirm the facilitation effect of HA on the transport of BMBC; (2) to rate the antagonistic effect of HA and ionic strength on BMBC movement; (3) to investigate the effect of different valences of cations on the mobility of BMBC.

## 2. Materials and Methods

### 2.1. Materials

The improved method to prepare BMBC based on Naghdi and Taheran [21] and Lyu and Gao [17] was described in detail in our previous work [38]. In brief, the pristine ramie (*Boehmeria nivea* (L.) Gaudich) biochar that was produced using a tube furnace (MXG 1200-40s, SH Micro-X, Shanghai, China) at 500 °C was added into a planetary ball mill machine (YXQM-4L, Changsha MITR Instrument, Hunan, China) and intermittently ground to prepare BMBC.

The particle size distribution, zeta potentials, specific surface areas, surface charge number, microscopic morphology, elemental composition, and oxygen-containing functional groups of BMBC or pristine biochar were measured using a dynamic light scattering (Zetasizer nano ZS90, Malvern Instruments, Malvern, UK), N_2_ adsorption isotherms based on the Brunauer−Emmett−Teller (BET) method (F-Sorb 2400, Gold APP Instrument Corporation, Beijing, China), the ammonium acetate compulsory displacement method, scanning electron microscopy (SEM) and energy dispersive spectrometer (EDS) (S-4800 Hitachi, Tokyo, Japan), and a FT-IR spectrometer (Nicolet iS10, Shanghai Precision instrument, Shanghai, China), respectively. 

The purified humic acid (HA) (Guangfu fine chemical research institute, Tianjin, China) was obtained through sequential dissolution in 0.1 M NaOH and 0.1 M HCl while adjusting the pH to remove the impurities, and then lyophilization. The stock solution of HA was prepared for the subsequent experiments.

BMBC stock solution (100 mg L^−1^) was mixed with a certain quality of BMBC powder with the prepared background electrolyte solution (with or without humic acid) and ultrasonicated using an ultrasonic cleaner (KQ-500DE, Kunshan Ultrasonic Instruments, Kunshan, China) for 2 h. The analytical pure NaCl, MgCl_2_, or AlCl_3_ and deionized (DI) water were used to obtain the background electrolyte solutions with corresponding ionic strength (IS).

The sieved granular medium (0.425–0.850 mm quartz sand, Zhouzhi County quartz sand factory, Zhouzhi, China) was successively soaked and washed with tap water, acid and alkali solutions, and DI water. After drying in an oven at 10 °C for 24 h, the cleaned sand was preserved for the column experiments. The zeta potentials of the quartz sand were determined using a Zeta-Plus analyzer (Zetasizer nano ZS90) [39]. 

### 2.2. Column Experiment

The cleaned quartz sand was wet-packed in a plexiglass column with a 15 cm length and 3 cm internal diameter. The porosity and the pore volume (PV) of the packed columns were approximately 0.42 and 45 mL, respectively. A steady flow from bottom to top with a velocity of 1.0 mL min^−1^ was achieved using a peristaltic pump (HL-2B, Shanghai Huxi Analytical Instrument Factory Co., Ltd., Shanghai, China). At least 3 PVs of background electrolyte solution (with or without HA) were utilized to stabilize the solution chemistry of the packed columns. Then, 3 PVs of the BMBC solutions with the objective HA concentrations (0, 1, 5 mg L^−1^), cation types (Na^+^, Mg^2+^, Al^3+^), and ISs, and more than 3 PVs of the corresponding background solutions were sequentially injected. It should be noted that the experiments were carried out under the condition of 7 ± 0.05 except for the part of Al^3+^ that was at a pH of 4, as the environmental chemistry and species of Al^3+^ speciation are complex [40]. The ISs of Na^+^ were 1, 10, and 100 mmol L^−1^ (mM), and those of Mg^2+^ and Al^3+^ were 0.1, 1, and 10 mM because of the consideration of the fast aggregation of BMBC under the effect of high-valence cations. The effluents were collected every 10 min using a fraction collector (BS-100A, Shanghai Huxi Analytical Instrument Factory Co., Ltd., Shanghai, China) and measured using a UV-VIS spectrometer (UV–2800, Unico Shanghai Instrument Co., Ltd., Shanghai, China) at 790 nm [34]. Our pre-experiments and previous studies manifested that the background electrolyte solution did not affect the determination of BMBC [32]. 

### 2.3. Mathematical Model

The transport of BMBC in the quartz sand columns was described using the advection-dispersion equations, and the parameters were obtained using HYDRUS-1D software [27,41]:

(1) $$\frac{\partial C_{w}}{\partial t}=D\frac{\partial^{2}C_{w}}{\partial z^{2}}-v\frac{\partial C_{w}}{\partial z}-\frac{\rho }{\theta }\frac{\partial S}{\partial t}$$

(2) $$\frac{\rho }{\theta }\frac{\partial S}{\partial t}=k\left(1-\frac{S}{S_{max}}\right)C_{w}$$

where *C_w_* is the concentration of BMBC (mg L^−1^); *D* is the dispersion coefficient (cm^2^ min^−1^); *v* is the Darcy velocity (cm min^−1^); *ρ* is the bulk density (g cm^−3^); *θ* is the porosity; *k* is the first-order retention coefficient (min^−1^); and *S_max_* is the maximum deposited concentration of BMBC (mg g^−1^). 

The estimation methods for the surface potentials of BMBC and granular media were based on diffuse double-layer theory and Gouy–Chapman theory [42,43]. Without considering ion polarization, BMBC and quartz sand in a 1:1 type of electrolyte solution (i.e., NaCl) can be calculated:

(3) $$\varphi_{0}=-\frac{2RT}{ZF}\ln\left(\frac{1-a}{1+a}\right)$$

(4) $$\frac{\kappa SCN}{Sc_{0}}=1+\frac{4}{1+a}-\frac{4}{1+e^{-1}a}$$

(5) $$\kappa =\sqrt{\frac{8\pi F^{2}Z^{2}c_{0}}{\varepsilon RT}}$$

where *φ*_0_ (V) is the surface potential, R (J mol^−1^ K^−1^) is the gas constant, T (K) is the absolute temperature, Z is the valence of the cation, F (C mol^−^^1^) is Faraday’s constant, *a* is the intermediate variable, SCN (cmol kg^−1^) is the surface charge number, *S* (m^2^ g^−1^) is the specific surface area, *κ* (dm^−1^) is the Debye–Hückel parameter, and *c*_0_ (mol L^−1^) is the equilibrium concentration of the cation in the bulk solution. 

The surface charge density *σ_0_* can also be calculated [44]:

(6) $$\sigma_{0}=\frac{F\;SCN}{10^{5}S}$$

where the unit of *σ*_0_ is C dm^−2^.

BMBC and quartz sand in a 2:1 type of electrolyte solution (i.e., MgCl_2_) can be calculated:

(7) $$\varphi_{0}=-\frac{RT}{F}\ln\frac{{\left(exp^{2b}+1\right)}^{2}}{{\left(exp^{2b}\right)}^{2}-exp^{2b}+1}$$

(8) $$\begin{array}{c} \frac{\kappa CEC}{SSAc_{0}}=1-\frac{3\left(2+\sqrt{3}\right)}{exp^{2b+1}-\left(2+\sqrt{3}\right)}+\frac{3\left(2+\sqrt{3}\right)}{exp^{2b}-\left(2+\sqrt{3}\right)}-\\ \frac{3\left(2-\sqrt{3}\right)}{exp^{2b+1}-\left(2-\sqrt{3}\right)}+\frac{3\left(2-\sqrt{3}\right)}{exp^{2b}-\left(2-\sqrt{3}\right)} \end{array}$$

where *b* is the intermediate variable.

## 3. Results and Discussion 

### 3.1. Properties of Ball-Milled Biochar, Pristine Biochar, and Quart Sand

The properties of the BMBC and raw biochar were described in our previous study [38]. In brief, the pristine biochar has a well-developed pore structure. The BMBC particles were fine and irregular after ball milling. More O-containing functional groups (e.g., hydroxyl, carboxyl) of biochar were exposed during the grinding process, while the elemental composition (C 73%, O 18%, and metal elements Ca 5%, Mg 1%, Al and K < 1%) was hardly changed. In addition, the specific surface area of BMBC was significantly increased compared to pristine biochar and reached 179.31 m^2^ g^−1^. The surface charge number of BMBC was 71.91 cmol kg^−1^. Correspondingly, the specific surface area and surface charge number of the quartz sand were 0.07 m^2^ g^−1^ and 0.6 cmol kg^−1^ [45], respectively.

Though it possessed a large surface charge number, the surface charge density of BMBC was 0.39 C m^−2^ because of the significant specific surface area. By contrast, the surface charge density of quartz sand was 0.83 C m^−2^. The high surface charge density contributed to a high absolute value of surface potentials (Table 1), and the surface potentials of BMBC or quartz sand were much more negative than the zeta potentials in Na^+^ or Mg^2+^ without HA. Furthermore, the difference in values between surface potentials and zeta potentials could reach one order of magnitude. 

### 3.2. HA-Facilitated Transport of BMBC

**Figure 1:**
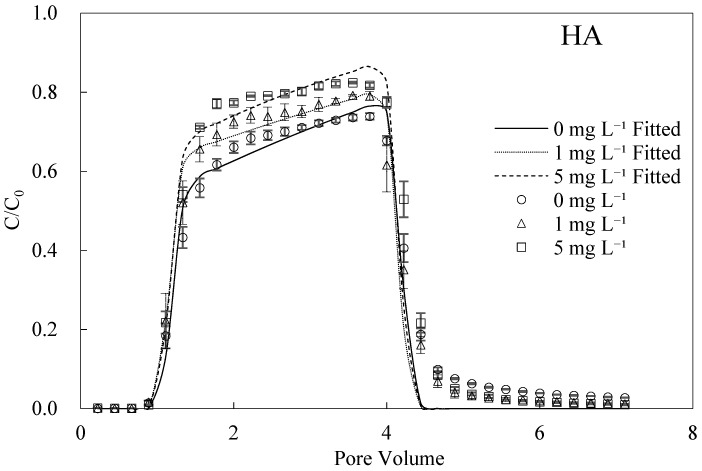
Breakthrough and fitting curves of BMBC at different concentrations of HA.

The breakthrough and fitting curves of BMBC are shown in Figure 1. The retention of BMBC in the packed columns decreased with the HA concentration. Under the condition without HA or cations, BMBC manifested a strong mobility and a slight tailing phenomenon in the elution process. As the concentration of HA increased, the maximum value of BMBC breakthrough curves increased and the BMBC concentrations declined at the tail. It can be obtained from Table 2 that when the HA concentrations were 0, 1, and 5 mg L^−1^, the corresponding recovery rates of BMBC were 74.29%, 76.89%, and 83.93% and the fitted *S_max_* decreased from 42.85 to 25.00 mg g^−1^. The same trend was maintained with the addition of the different cations (Na^+^, Mg^2+^, and Al^3+^) in most cases. Moreover, the presence of HA retarded the ripening phenomenon caused by 100 mM Na^+^, and maintained the transport of BMBC at 5 mg L^−1^. However, the facilitated effect of HA hardly functioned at 10 mM Mg^2^.

The effect of organic matter on the movement of nanoparticles or colloids has been specifically investigated, and it still functioned in the transport of BMBC [29,34,35,46]. The mechanisms of HA could be concluded in 2 parts: (1) the steric effect prevented the attachment of BMBC onto the medium surface; (2) the electrostatic repulsion was enhanced between BMBC and quartz sand because of the adsorption of HA, thus stabilizing BMBC in solutions [32,36]. Our observed results also demonstrated that HA increased the zeta potentials (absolute values) of BMBC and quartz sand. In particular, the zeta potential of BMBC decreased from −28.36 to −31.57 mV, and that of sand decreased from −47.10 to −49.95 mV, which indicated that the stability of BMBC was improved and led to a decline in BMBC retention. However, there was an upper limit of the facilitated effect from HA. When the HA concentration increased to a specific value (usually above 4 mg L^−1^), the zeta potentials of particles were not significantly increased [32,35]. The limited increase in zeta potential at 1 mg L^−1^ HA might be attributed to the high surface charge density of quartz sand, thus weakening the effect from HA. Actually, it has been reported that the concentration of dissolved organic carbon in aquatic ecosystems ranged from 0.1 to 10 mg L^−1^ [47]. Therefore, the facilitated effect of HA as a model of organic matter concluded from the previous studies might be overrated. 

### 3.3. Antagonistic Effect of Humic Acid and Ionic Strength in the Presence of Na^+^ and Mg^2+^

**Figure 2:**
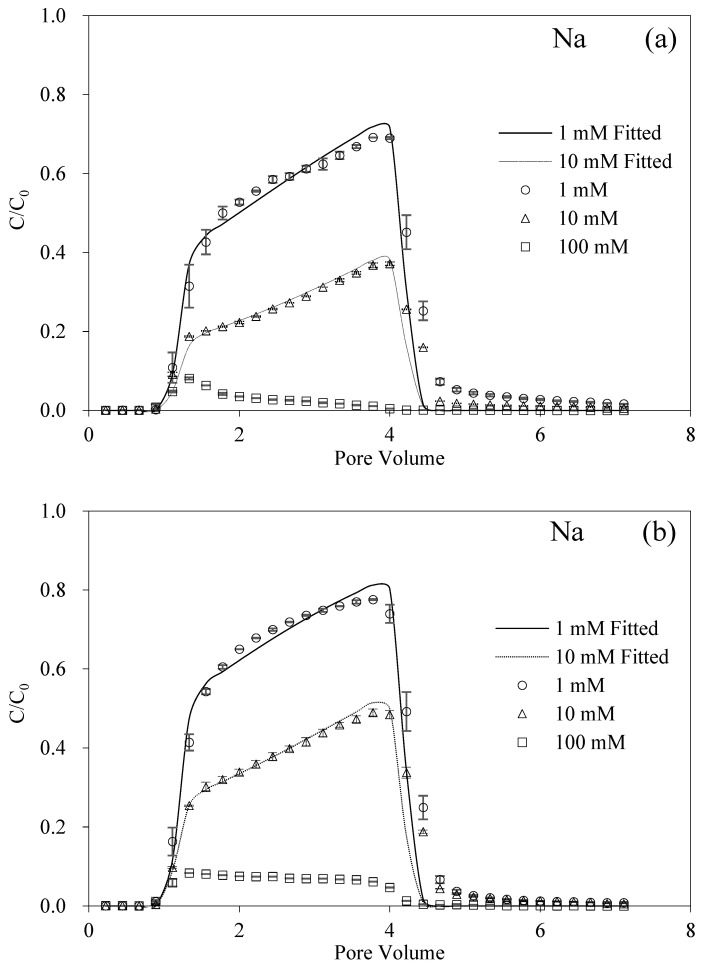

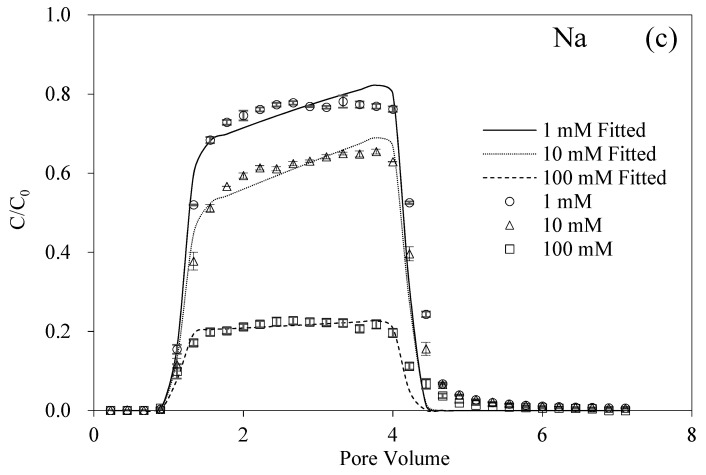
Breakthrough and fitting curves of BMBC at different ISs of Na with 0 (**a**), 1 (**b**), and 5 (**c**) mg L^−1^ HA.

Our results demonstrated that the increased HA facilitated the transport of BMBC, and that the increased IS reduced the mobility of BMBC. As manifested in Figure 2a, the effect of different ISs of Na^+^ on the transport of BMBC is unsurprisingly similar to previous research [28,31]. With the IS increased in the background electrolyte solutions, the retention of BMBC in the packed column also increased. Compared with Figure 2a–c, the presence of HA enhanced the mobility of BMBC, although the effect from HA at 1 mM was limited. It can be seen from Table 1 that after the addition of HA (1.5 mg g^−1^), the recovery rates of BMBC increased from 64.06% to 74.03% and 79.79%. The most significant promoting effect of HA was under the IS of 10 mM. On the other hand, BMBC experienced a slow penetration process at HA concentrations of 0 or 1 mg L^−1^. Specifically, its breakthrough curve slowly climbed to the maximum value. When the HA concentration was 5 mg g^−1^, the penetration process of BMBC was greatly accelerated and the recoveries significantly rose. Moreover, the ripening phenomenon that occurred in the IS of 100 mM disappeared and BMBC passed through the quartz sand column and maintained low normalized concentrations (approximate 0.2) with the “low platform” type of breakthrough curve.

For the monovalent Na^+^, when the IS of the background solution was increased from 1 to 100 mM, the recovery of BMBC decreased from 64.06% to 3.39%, and the retention amount in the sand columns also increased dramatically. The shape change of the breakthrough curves of BMBC in the increasing IS represented the transport mechanism that the particle–particle attachment tendency was higher than the particle–surface attachment tendency [48]. Such a high IS compressed the electrical double layer of BMBC based on Equation (5), which decreased the repulsive interaction between particles so that aggregation occurred, while, with the addition of HA, the recoveries of BMBC increased from 3.39% to 7.56% and 23.31%. In particular, the breakthrough curve of BMBC in 100 mM and 5 mg L^−1^ HA obtained the maximum *S_max_* value of 690.07 mg g^−1^ and manifested that the leading factor was the mechanism of irreversible deposition [49]. 

**Figure 3:**
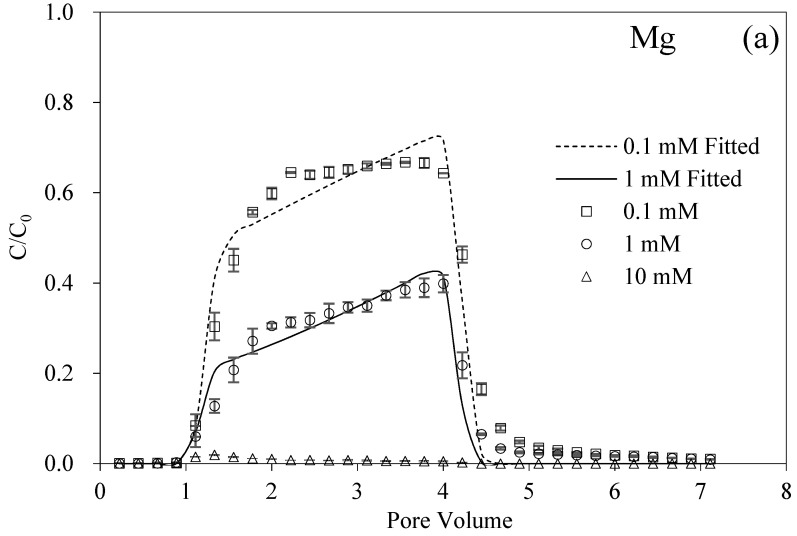

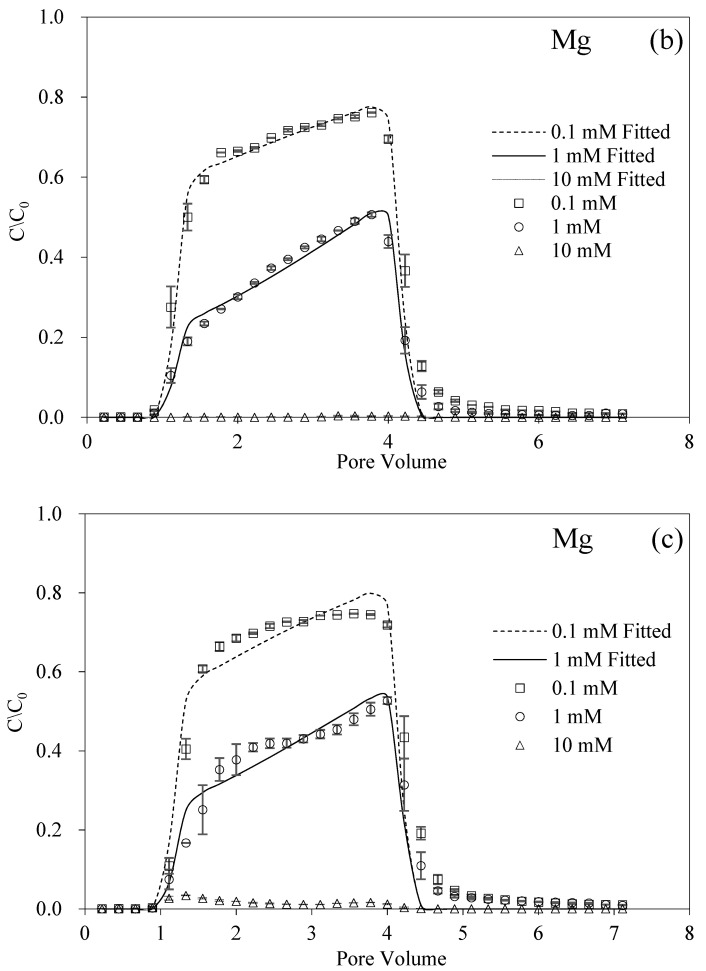
Breakthrough and fitting curves of BMBC at different ISs of Mg with 0 (**a**), 1 (**b**), and 5 (**c**) mg L^−1^ HA.

Figure 3a showed the effect of different MgCl_2_IS on the transport of BMBC compared to Na^+^. It should be noted that the valence of counterions strongly affected the stability of colloids, and the concentrations of MgCl_2_ and AlCl_3_ were lower than that of NaCl (NaCl: MgCl_2_: AlCl_3_ = 6:3:1) at the same IS based on the Shulze–Hardy rule [50]. For MgCl_2_, as the IS increased from 0.1 mM to 10 mM, the overall level of the normalized concentrations of BMBC decreased in turn, and the recovery rates were also significantly reduced from 65.37% to 0.98%. Meanwhile, as a comparison, the addition of HA facilitated the transport of BMBC. At 10 mM, though the recovery of BMBC increased, the *S_max_* value changed from 107.60 to 79.64 and 79.55 mg g^−1^. The above results indicated that the antagonistic effect of HA and IS on the movement of BMBC in porous media functioned for both Na^+^ and Mg^2+^ except for the IS of MgCl_2_ 10 mM.

The response of BMBC to the divalent cation was more sensitive than that of the monovalent cation. Almost all the BMBC was retained in the quartz columns at 10 mM of Mg^2+^. The different mechanism of Mg^2+^ and Na^+^ on the mobility of BMBC was mainly attributed to the bridging effect of Mg^2+^ [51,52]. On the other hand, the critical coagulation concentrations (CCCs) of biochar colloids in divalent cation solutions (Ca^2+^, 38 mM) were much lower than those in monovalent cation solutions (Na^+^, 183 mM) [32]. It can be found in Figure 2 and Figure 3 that the mobility of BMBC in Mg^2+^ was significantly weakened than those in Na^+^ at ISs of 1 and 10 mM. As shown in Table 2, the theoretical surface potentials of BMBC and quartz sand in Mg^2+^ 10 mM without HA were −121.75 mV and −208.72 mV, respectively, while the observed zeta potentials of BMBC were reversed. The values were +7.59 mV without HA and +6.83 mV in 1 mg L^−1^ HA. The charge reversal originated from the nonelectrostatic ion–surface dispersion interactions and the ion correlation forces, and the effect of charge reversal generated from Mg^2+^ was more significant than other divalent cations (Ca^2+^ > Sr^2+^ > Ba^2+^, a Hofmeister series) [53]. The calculated surface potentials and zeta potentials of the medium were negative due to a higher surface charge density. Therefore, the differences in electrokinetic properties between BMBC and quartz sand and the instability of BMBC presented as low zeta potentials resulted in the retention of BMBC in the packed columns. In addition, the commonly existing electrostatic shielding effect of cations undermined the electronegativity of BMBC particles and the surface of the medium [50,54]. The combination of the bridging effect and charge shielding effect led to the “permanent” deposition of BMBC particles so that the high concentration of HA could not promote the transport of BMBC at 10 mM.

The calculated surface potentials of BMBC and granular media in Na^+^ and Mg^2+^ solutions are shown in Table 2. Note that the surface potential in the presence of HA was not given, because of the limitation of the theoretical arithmetic. In this study, the DLVO theory was not to analyze the interaction between BMBC and the granular media, because the neglection of the specific ion effect and the replacement of surface potential with the zeta potential representing the electric potential of the shear plane that was far away from the solid–liquid interface at the dilute electrolyte solution, therefore, might lead to indirect results [55,56,57]. HA stabilized the BMBC particles in solution so that promoted the transport of BMBC in porous media. HA contained different types of functional groups, mainly phenolic and carboxyl groups, and the deprotonation of these groups produced a repulsive force that caused the stretched chain structure [36]. 

Generally, the oxygen-containing functional groups on the surface of HA made it a higher surface charge density. The attachment to the surface of BMBC and quartz sand could increase the charge density and enhance the steric effect, thus improving the stability and mobility of BMBC [58]. Although HA usually was taken as a model of organic matter in experiments, there were still many differences between HA and natural organic matter. Particularly, the ISs of dissolved organic matter extracted from different sources were significantly larger than that of HA [35]. Therefore, the limited facilitated effect on the transport of nanoparticles might be attributed to the discrepancies of ISs. The antagonism and balance between IS and HA on the fate and transport of BMBC could reveal the mechanism of how organic matter and solution chemistry influence the environmental implication of nanoparticles.

### 3.4. Effect of Al^3+^ and HA on Transport of BMBC under Acidic Conditions

**Figure 4:**
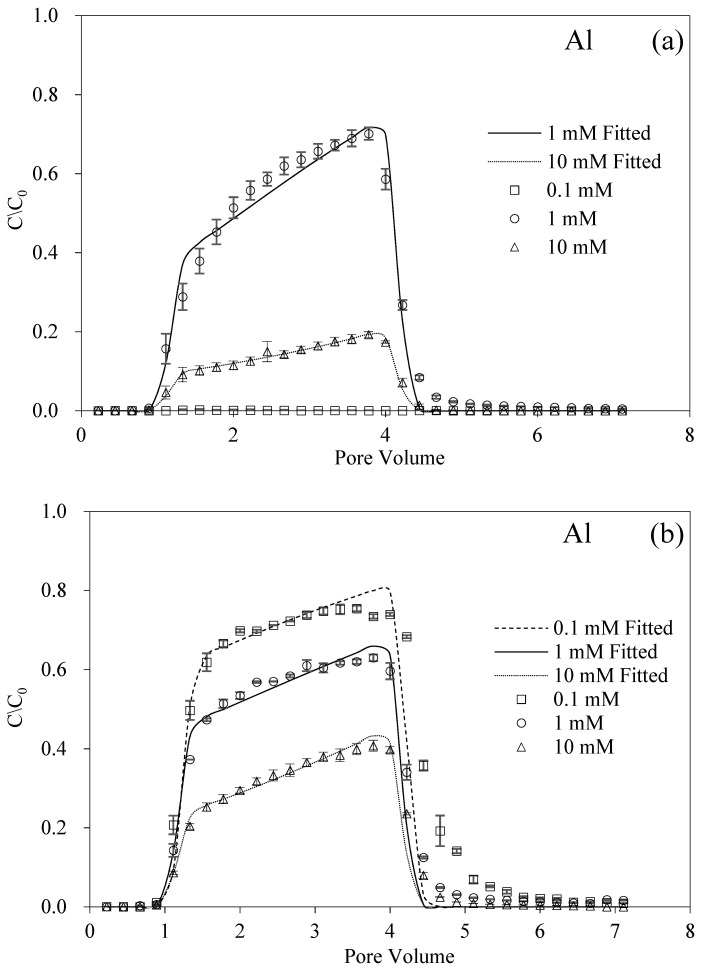

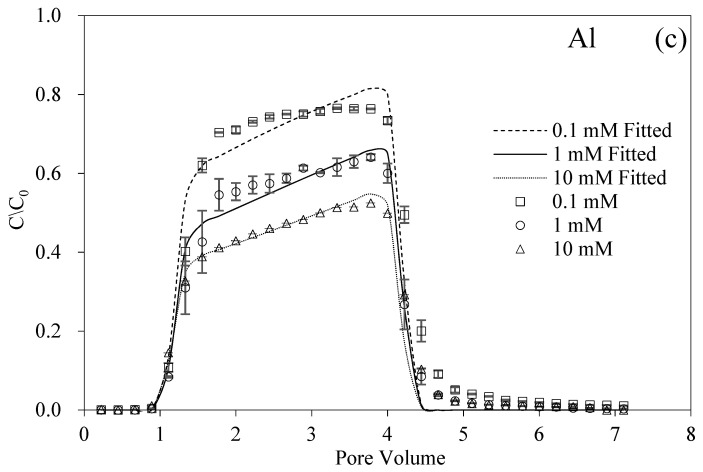
Breakthrough and fitting curves of BMBC at different ISs of Al with 0 (**a**), 1 (**b**), and 5 (**c**) mg L^−1^ HA.

The experimentally related pH of Al^3+^ was determined under acidic conditions due to the solubility of Al^3+^ [40]. Therefore, it should be noted that this part of the experimental design was relatively independent of the other parts. The results could appropriately extend the application of BMBC to the proper environment. Figure 4a shows the transport of BMBC affected by Al^3+^ under acidic conditions. The results indicated that Al^3+^ with an IS of 0.1 mM directly inhibited the transport of BMBC. In particular, only 0.25% of BMBC passed through the quartz sand column, and most of the BMBC was deposited on the bottom of the sand column. Interestingly, with IS increased, BMBC passed through the packed columns instead of retention. 

The advection-dispersion equations could also well describe the transport of BMBC under the effect of Al^3+^ and HA. Except for the unfavorable condition of 0.1 mM, the recoveries of BMBC were 59.35% and 14.97% and the *S_max_* values were 47.09 mg g^−1^ and 189.80 mg g^−1^ at ISs of 1 mM and 10 mM, respectively. From recovery rates that were approximately 60%, while the addition of HA in IS 1 mM did not influence the mobility of BMBC, the fitted parameters, however, sequentially were 47.09, 62.49, and 55.37 mg g^−1^ at 1 mM from HA 0–5 mg L^−1^. The difference was also reflected in the breakthrough curves. In particular, these curves went from “sharp” (rapid rise) to more “smooth” (asymmetric rise in the plateau), and the maximum normalized concentration of BMBC changed from 0.70 to 0.63 and 0.64 with the addition of HA, which represented a typical detachment decline process, and the simulated *k* values of BMBC decreased from 2.10 × 10^−2^ to 1.72 × 10^−2^ and 1.71 × 10^−2^ min^−1^ [48]. It could be concluded that the existence of HA influenced mechanically the transport of BMBC. Furthermore, it was reported that the effect of HA easily reached the upper limit (especially over 4 mg L^−1^), which was more obvious for considering the fitted *S_max_* values of BMBC significantly decreased from 189.80 to 99.46 and 89.82 mg g^−1^ [35].

The trivalent cation (i.e., Al^3+^) presented a more significant bridging effect than the divalent cation. Actually, the presence of Al^3+^ significantly influenced the stability of BMBC [51]. The stability of colloid particles in the aqueous solution and the degree of repulsive electrostatic interaction between the adjacent charged particles can be reflected by the zeta potentials. Our pre-experiments indicated that the isoelectric points of BMBC and quartz sand were both below pH 4. Therefore, their zeta potentials were both negative without the effect of Al^3+^. However, the observed zeta potentials of BMBC (+4.50 mV) and quartz sand (−9.50 mV) were totally contrary, which signified the opposite direction in the electrophoresis. That is to say, the BMBC has a strong tendency to deposit on quartz sand under the effect of the electrostatic interaction. With the increase in the IS, the reverse surface charge was observed again. Moreover, the zeta potentials of the quartz sand with a larger surface charge density than BMBC turned positive under 1 and 10 mM conditions. The observed values of BMBC and the medium were +21.70 mV and +14.89 mV at IS of 1 mM, respectively. From this perspective, BMBC became more stable in the solution. In the previous research, the surface charge reversal for quartz sand from negative to positive yielded by Al^3+^ was also reported [59]. The electrostatic repulsion predominated the interaction between the positively charged BMBC and quartz sand, thus enhancing the mobility of BMBC. With the IS of AlCl_3_ increased to 10 mM, the recovery of BMBC decreasing from 59.35% to 14.97% might be attributed to the enhancement of hydrophobicity. It was reported that the contact angle and the sign of hydrophobicity increased and then declined with the Al^3+^ concentrations increasing from 0 to 10 and 40 mg L^−1^ [60]. 

For the different ISs of Al^3+^, the presence of HA could influence the transport of BMBC in porous media, as shown in Figure 4b,c. The breakthrough curves of BMBC shared the same pattern that the normalized concentrations rapidly increased and then reached the maximum value relatively slowly, which demonstrated that the particle–solid association was temporarily retained and the reversible deposition therefore detached [48]. The solution chemistry of low pH could enhance the affinity of dissolved organic matter (i.e., HA) to BMBC under the effect of electrostatic interaction [47,58]. As a consequence, the zeta potentials of BMBC became more negative with the adsorption of HA. On the other hand, the strong binding sites on HA were occupied and the binding affinity from inducing conformational changes of the humic macromolecule was reduced, which made binding sites unavailable [61,62]. The limited effect from the dilute concentration of Al^3+^ could not cover the facilitated effect of HA. Therefore, the addition of HA restored the mobility of BMBC in porous media.

The facilitated effect of HA and the inhibitory effect of IS on the transport of BMBC have functioned in most cases. This antagonism, when the HA concentration was high, counteracted the effect of IS on BMBC, thereby promoting the transport of BMBC in the medium. Moreover, the protonation of HA O-containing functional groups was enhanced under acidic conditions [63]. However, the increase in cation concentration could enhance the shielding in the negative surface charge of BMBC or the medium, or even reverse the charged surface [53,59]. The larger the amount of charge, the greater the effect of cations on the formation of pseudo-capillaries [36].

The results demonstrated that the limited facilitated effect of natural organic matter might be the result of the interaction between the dissolved substance and the coexisting cations.

## 4. Conclusions 

In this research, the effect of cation type, IS, and HA was systematically studied using the packed sand column experiments. The results demonstrated that the presence of HA enhanced the mobility of BMBC in most cases by the negatively charged surface and steric effect and that the enhancement effect of HA had an upper limit. The increased IS, however, inhibited the facilitated effect from HA. The antagonistic effect of HA and IS on the transport of BMBC functioned in most cases, where the presence of one could weaken the effect from the other. The increased ISs of monovalent cation Na^+^ mainly influenced the transport of BMBC by compression of the electrical double layer, leading to the decrease in the electrostatic interaction, while the divalent cation Mg^2+^ and trivalent cation Al^3+^ were largely attributed to the bridging effect. When the surface charge reverse occurred in MgCl_2_ IS 10 mM, the permanent deposition of BMBC invalidated the facilitated effect of HA. The observation of reverse charge was more obvious in Al^3+^ under acidic conditions, which resulted in the restabilization and transport of BMBC in an IS of 1 mM. It indicated that the mobility of engineering material might be facilitated in the presence of Al^3+^, especially under acidic conditions. The fitted results of advection-dispersion equations indicated that the transport mechanism of BMBC in porous media was mainly particle–surface attachment. Though this work systematically investigated the transport of BMBC in porous media, the abundance of carbon-based engineering materials and the heterogeneity of the underground environment still need further laboratory and field work to promote environmental protection and sustainable development.

## Figures and Tables

**Table 1:** Theoretical surface potential of BMBC and quartz sand.

	IS (mM)	*φ*_0-_*_BMBC_* (mV)	*φ*_0-_*_Q_* (mV)
Na	1	−274.06	−431.35
	10	−215.78	−371.95
	100	−159.04	−312.88
Mg	0.1	−180.49	−267.41
	1	−151.05	−237.82
	10	−121.75	−208.72

**Table 2:** Experimental conditions and fitting results for all column experiments.

Cation Type	HA (mg L^−1^)	IS (mM)	ζ_BMBC_ (mV)	*ζ_Q_* (mV)	*k* (min^−1^)	*S_max_* (mg g^−1^)	R^2^	Recovery (%)
/	0	/	−28.36	−47.10	1.23 × 10^−2^	42.85	0.98	74.29
	1		−29.92	−46.57	9.99 × 10^−3^	39.43	0.98	76.89
	5		−31.57	−49.95	8.82 × 10^−3^	25.00	0.97	83.93
Na	0	1	−25.69	−45.47	1.81 × 10^−2^	46.64	0.98	64.06
		10	−22.52	−34.50	3.66 × 10^−2^	110.54	0.95	31.60
		100	−4.60	−26.48	/	/	/	3.39
	1	1	−27.21	−47.92	1.37 × 10^−2^	33.22	0.98	74.03
		10	−23.07	−38.61	2.85 × 10^−2^	83.78	0.96	43.96
		100	−6.49	−27.55	/	/	/	7.56
	5	1	−28.47	−48.42	8.89 × 10^−3^	32.81	0.97	79.79
		10	−26.86	−39.23	1.47 × 10^−2^	57.96	0.98	64.15
		100	−11.22	−29.83	3.52 × 10^−2^	690.07	0.97	23.31
Mg	0	0.1	−25.87	−40.70	1.52 × 10^−2^	49.37	0.98	65.37
		1	−14.94	−32.50	3.42 × 10^−2^	107.60	0.97	34.63
		10	+7.59	−25.08	/	/	/	0.98
	1	0.1	−25.56	−41.43	1.14 × 10^−2^	41.93	0.99	73.93
		1	−15.80	−34.09	3.32 × 10^−2^	79.64	0.99	39.74
		10	+6.83	−26.64	/	/	/	0.23
	5	0.1	−26.57	−39.70	1.29 × 10^−2^	36.01	0.97	73.83
		1	−15.61	−35.56	2.81 × 10^−2^	79.55	0.97	44.39
		10	−2.30	−26.35	/	/	/	1.91
Al	0	0.1	+4.50	−9.50	/	/	/	0.25
		1	+21.70	+14.89	2.10 × 10^−2^	47.09	0.99	59.35
		10	+33.74	+17.20	5.12 × 10^−2^	189.80	0.99	14.97
	1	0.1	−8.27	−12.31	1.00 × 10^−2^	35.41	0.94	81.03
		1	+17.98	+17.67	1.72 × 10^−2^	62.49	0.98	60.27
		10	+26.25	+19.08	3.15 × 10^−2^	99.46	0.98	35.81
	5	0.1	−15.42	−16.31	1.13 × 10^−2^	33.52	0.97	76.71
		1	−13.69	−22.68	1.71 × 10^−2^	55.37	0.99	58.22
		10	+16.74	+19.94	2.16 × 10^−2^	89.82	0.99	49.44

ζ_BMBC_—zeta potential of BMBC; ζ_Q_—zeta potential of quartz sand; *k*—first-order retention coefficient; *S_max_*—the maximum deposited particle concentration.

## Data Availability

Not applicable.

## References

[1] Marris E. (2006). Putting the carbon back: Black is the new green. Nature.

[2] Kleiner K. (2009). The bright prospect of biochar. Nat. Rep. Clim. Change.

[3] Chen W., Meng J., Han X., Lan Y., Zhang W. (2019). Past, present, and future of biochar. Biochar.

[4] Razzaghi F., Obour P.B., Arthur E. (2020). Does biochar improve soil water retention? A systematic review and meta-analysis. Geoderma.

[5] Godlewska P., Schmidt H.P., Ok Y.S., Oleszczuk P. (2017). Biochar for composting improvement and contaminants reduction. A review. Bioresour. Technol..

[6] Ibrahim H.M., Al-Wabel M.I., Usman A.R., Al-Omran A. (2013). Effect of Conocarpus biochar application on the hydraulic properties of a sandy loam soil. Soil Sci. Soc. Am. J..

[7] El-Naggar A., Lee S.S., Rinklebe J., Farooq M., Song H., Sarmah A.K., Zimmerman A.R., Ahmad M., Shaheen S.M., Ok Y.S. (2019). Biochar application to low fertility soils: A review of current status, and future prospects. Geoderma.

[8] Inyang M., Gao B., Yao Y., Xue Y., Zimmerman A.R., Mosa A., Pullammanappallil P., Ok Y.S., Cao X. (2016). A review of biochar as a low-cost adsorbent for aqueous heavy metal removal. Crit. Rev. Environ. Sci. Technol..

[9] Ahmed M.B., Zhou J.L., Ngo H.H., Guo W., Chen M. (2016). Progress in the preparation and application of modified biochar for improved contaminant removal from water and wastewater. Bioresour. Technol..

[10] Srivastav A.L., Pham T.D., Izah S.C., Singh N., Singh P.K. (2021). Biochar Adsorbents for Arsenic Removal from Water Environment: A Review. Bull. Environ. Contam. Toxicol..

[11] Liu G., Zheng H., Jiang Z., Zhao J., Wang Z., Pan B., Xing B. (2018). Formation and Physicochemical Characteristics of Nano Biochar: Insight into Chemical and Colloidal Stability. Environ. Sci. Technol..

[12] Li C., Bair D.A., Parikh S.J. (2018). Estimating potential dust emissions from biochar amended soils under simulated tillage. Sci. Total Environ..

[13] Yin X., Yu L., Luo X., Zhang Z., Sun H., Mosa A.A., Wang N. (2019). Sorption of Pb (II) onto <1 μm effective diameter clay minerals extracted from different soils of the Loess Plateau, China. Geoderma.

[14] Peterson S.C., Jackson M.A., Kim S., Palmquist D.E. (2012). Increasing biochar surface area: Optimization of ball milling parameters. Powder Technol..

[15] Delogu F., Gorrasi G., Sorrentino A. (2017). Fabrication of polymer nanocomposites via ball milling: Present status and future perspectives. Prog. Mater. Sci..

[16] Amusat S.O., Kebede T.G., Dube S., Nindi M.M. (2021). Ball-milling synthesis of biochar and biochar–based nanocomposites and prospects for removal of emerging contaminants: A review. J. Water Process Eng..

[17] Lyu H., Gao B., He F., Zimmerman A.R., Ding C., Huang H., Tang J. (2018). Effects of ball milling on the physicochemical and sorptive properties of biochar: Experimental observations and governing mechanisms. Environ. Pollut..

[18] Wang B., Gao B., Wan Y. (2018). Entrapment of ball-milled biochar in Ca-alginate beads for the removal of aqueous Cd(II). J. Ind. Eng. Chem..

[19] Lyu H., Xia S., Tang J., Zhang Y., Gao B., Shen B. (2020). Thiol-modified biochar synthesized by a facile ball-milling method for enhanced sorption of inorganic Hg^2+^ and organic CH_3_Hg^+^. J. Hazard. Mater..

[20] Zhang Q., Wang J., Lyu H., Zhao Q., Jiang L., Liu L. (2019). Ball-milled biochar for galaxolide removal: Sorption performance and governing mechanisms. Sci. Total Environ..

[21] Naghdi M., Taheran M., Brar S.K., Rouissi T., Verma M.P., Surampalli R.Y., Valero J.R. (2017). A green method for production of nanobiochar by ball milling- optimization and characterization. J. Clean. Prod..

[22] Lyu H., Gao B., He F., Zimmerman A.R., Ding C., Tang J., Crittenden J.C. (2018). Experimental and modeling investigations of ball-milled biochar for the removal of aqueous methylene blue. Chem. Eng. J..

[23] Xiang W., Wan Y., Zhang X., Tan Z., Xia T., Zheng Y., Gao B. (2020). Adsorption of tetracycline hydrochloride onto ball-milled biochar: Governing factors and mechanisms. Chemosphere.

[24] Lægdsmand M., Villholth K.G., Ullum M., Jensen K.H. (1999). Processes of colloid mobilization and transport in macroporous soil monoliths. Geoderma.

[25] Liu X., Tang J., Wang L., Liu Q., Liu R. (2019). A comparative analysis of ball-milled biochar, graphene oxide, and multi-walled carbon nanotubes with respect to toxicity induction in Streptomyces. J. Environ. Manag..

[26] Liu X., Tang J., Wang L., Liu R. (2020). Synergistic toxic effects of ball-milled biochar and copper oxide nanoparticles on Streptomyces coelicolor M145. Sci. Total Environ..

[27] Zhang W., Niu J., Morales V.L., Chen X., Hay A.G., Lehmann J., Steenhuis T.S. (2010). Transport and retention of biochar particles in porous media: Effect of pH, ionic strength, and particle size. Ecohydrology.

[28] Wang D., Zhang W., Hao X., Zhou D. (2013). Transport of Biochar Particles in Saturated Granular Media: Effects of Pyrolysis Temperature and Particle Size. Environ. Sci. Technol..

[29] Wang D., Zhang W., Zhou D. (2013). Antagonistic Effects of Humic Acid and Iron Oxyhydroxide Grain-Coating on Biochar Nanoparticle Transport in Saturated Sand. Environ. Sci. Technol..

[30] Wang M., Gao B., Tang D. (2016). Review of key factors controlling engineered nanoparticle transport in porous media. J. Hazard. Mater..

[31] Yang W., Wang Y., Sharma P., Li B., Liu K., Liu J., Flury M., Shang J. (2017). Effect of naphthalene on transport and retention of biochar colloids through saturated porous media. Colloids Surf. A Physicochem. Eng. Asp..

[32] Yang W., Shang J., Sharma P., Li B., Liu K., Flury M. (2019). Colloidal stability and aggregation kinetics of biochar colloids: Effects of pyrolysis temperature, cation type, and humic acid concentrations. Sci. Total Environ..

[33] Song B., Chen M., Zhao L., Qiu H., Cao X. (2019). Physicochemical property and colloidal stability of micron- and nano-particle biochar derived from a variety of feedstock sources. Sci. Total Environ..

[34] Yang W., Wang Y., Shang J., Liu K., Sharma P., Liu J., Li B. (2017). Antagonistic effect of humic acid and naphthalene on biochar colloid transport in saturated porous media. Chemosphere.

[35] Zhang R., Zhang H., Tu C., Luo Y. (2019). The limited facilitating effect of dissolved organic matter extracted from organic wastes on the transport of titanium dioxide nanoparticles in acidic saturated porous media. Chemosphere.

[36] de Melo B.A.G., Motta F.L., Santana M.H.A. (2016). Humic acids: Structural properties and multiple functionalities for novel technological developments. Mater. Sci. Eng. C.

[37] Pham H.D., Dang T.H.M., Nguyen T.T.N., Nguyen T.A.H., Pham T.N.M., Pham T.D. (2021). Separation and determination of alkyl sulfate surfactants in wastewater by capillary electrophoresis coupled with contactless conductivity detection after preconcentration by simultaneous adsorption using alumina beads. Electrophoresis.

[38] Cao G., Sun J., Chen M., Sun H., Zhang G. (2021). Co-transport of ball-milled biochar and Cd2+ in saturated porous media. J. Hazard. Mater..

[39] Jiang Y., Zhang X., Yin X., Sun H., Wang N. (2018). Graphene oxide-facilitated transport of Pb^2+^ and Cd^2+^ in saturated porous media. Sci. Total Environ..

[40] Ščančar J., Milačič R. (2006). Aluminium speciation in environmental samples: A review. Anal. Bioanal. Chem..

[41] Jiang Y., Guan D., Liu Y., Yin X., Zhou S., Zhang G., Wang N., Sun H. (2020). The transport of graphitic carbon nitride in saturated porous media: Effect of hydrodynamic and solution chemistry. Chemosphere.

[42] Li H., Qing C.L., Wei S.Q., Jiang X.J. (2004). An approach to the method for determination of surface potential on solid/liquid interface: Theory. J. Colloid Interface Sci..

[43] Li S., Li H., Xu C.-Y., Huang X.-R., Xie D.-T., Ni J.-P. (2013). Particle Interaction Forces Induce Soil Particle Transport during Rainfall. Soil Sci. Soc. Am. J..

[44] Li H., Hou J., Liu X.M., Li R., Zhu H.L., Wu L.S. (2011). Combined Determination of Specific Surface Area and Surface Charge Properties of Charged Particles from a Single Experiment. Soil Sci. Soc. Am. J..

[45] Zhuang J., Flury M., Jin Y. (2003). Colloid-Facilitated Cs Transport through Water-Saturated Hanford Sediment and Ottawa Sand. Environ. Sci. Technol..

[46] Lv X., Gao B., Sun Y., Shi X., Xu H., Wu J. (2014). Effects of Humic Acid and Solution Chemistry on the Retention and Transport of Cerium Dioxide Nanoparticles in Saturated Porous Media. Water Air Soil Pollut..

[47] Yu S., Liu J., Yin Y., Shen M. (2018). Interactions between engineered nanoparticles and dissolved organic matter: A review on mechanisms and environmental effects. J. Environ. Sci..

[48] Babakhani P., Bridge J., Doong R.-A., Phenrat T. (2017). Continuum-based models and concepts for the transport of nanoparticles in saturated porous media: A state-of-the-science review. Adv. Colloid Interface Sci..

[49] Babakhani P., Fagerlund F., Shamsai A., Lowry G.V., Phenrat T. (2018). Modified MODFLOW-based model for simulating the agglomeration and transport of polymer-modified Fe0 nanoparticles in saturated porous media. Environ. Sci. Pollut. Res..

[50] Xu F., Wei C., Zeng Q., Li X., Alvarez P.J.J., Li Q., Qu X., Zhu D. (2017). Aggregation Behavior of Dissolved Black Carbon: Implications for Vertical Mass Flux and Fractionation in Aquatic Systems. Environ. Sci. Technol..

[51] Wang M., Gao B., Tang D., Yu C. (2018). Concurrent aggregation and transport of graphene oxide in saturated porous media: Roles of temperature, cation type, and electrolyte concentration. Environ. Pollut..

[52] Castan S., Sigmund G., Hüffer T., Hofmann T. (2019). Biochar particle aggregation in soil pore water: The influence of ionic strength and interactions with pyrene. Environ. Sci. Processes Impacts.

[53] Parsons D.F., Ninham B.W. (2010). Charge Reversal of Surfaces in Divalent Electrolytes: The Role of Ionic Dispersion Interactions. Langmuir.

[54] Wu L., Liu L., Gao B., Muñoz-Carpena R., Zhang M., Chen H., Zhou Z., Wang H. (2013). Aggregation Kinetics of Graphene Oxides in Aqueous Solutions: Experiments, Mechanisms, and Modeling. Langmuir.

[55] Hou J., Li H., Zhu H., Wu L. (2009). Determination of Clay Surface Potential: A More Reliable Approach. Soil Sci. Soc. Am. J..

[56] Li H., Wei S., Qing C., Yang J. (2003). Discussion on the position of the shear plane. J. Colloid Interface Sci..

[57] Du W., Liu X.M., Tian R., Li R., Ding W.Q., Li H. (2020). Specific ion effects of incomplete ion-exchange by electric field-induced ion polarization. Rsc Adv..

[58] Hu J.-D., Zevi Y., Kou X.-M., Xiao J., Wang X.-J., Jin Y. (2010). Effect of dissolved organic matter on the stability of magnetite nanoparticles under different pH and ionic strength conditions. Sci. Total Environ..

[59] Yukselen-Aksoy Y., Kaya A. (2011). A study of factors affecting on the zeta potential of kaolinite and quartz powder. Environ. Earth Sci..

[60] Zhang L., Zheng J., Tian S., Zhang H., Guan X., Zhu S., Zhang X., Bai Y., Xu P., Zhang J. (2020). Effects of Al^3+^ on the microstructure and bioflocculation of anoxic sludge. J. Environ. Sci..

[61] Buschmann J., Kappeler A., Lindauer U., Kistler D., Berg M., Sigg L. (2006). Arsenite and Arsenate Binding to Dissolved Humic Acids:  Influence of pH, Type of Humic Acid, and Aluminum. Environ. Sci. Technol..

[62] Yin X., Jiang Y., Tan Y., Meng X., Sun H., Wang N. (2019). Co-transport of graphene oxide and heavy metal ions in surface-modified porous media. Chemosphere.

[63] von Wandruszka R. (2000). Humic acids: Their detergent qualities and potential uses in pollution remediation. Geochem. Trans..

